# Learning in the moment: simulated patients’ engagement in students’ meaningful learning during communication training—a stimulated recall study

**DOI:** 10.1186/s41077-025-00370-2

**Published:** 2025-09-26

**Authors:** Annelies Lovink, Marleen Groenier, Anneke van der Niet, Jan-Joost Rethans, Walther van Mook

**Affiliations:** 1 Department of Technical Medicine, University of Twente, Hallenweg 5, Enschede, 75522 NH the Netherlands; 2 Department IQ Healthcare, Radboud University Medical Center, Nijmegen, the Netherlands; 3 Skillslab, Faculty of Health, Medicine and Life Sciences, Maastricht University, Maastricht, the Netherlands; 4 Department of Intensive Care Medicine, MUMC+, Maastricht, the Netherlands; 5 Academy for Postgraduate Medical Training, MUMC+, Maastricht, the Netherlands; 6 School of Health Professions Education, Maastricht University, Maastricht, The Netherlands

**Keywords:** Simulated patients (SPs), Medical communication, Student learning, Stimulated recall

## Abstract

**Background:**

Previous studies have focused on the role of simulated patient (SP) feedback on students’ learning outcomes *after* an SP-student encounter, whereas more recent studies have aimed to unravel meaningful learning *during* the encounter. We gain a more detailed understanding of students’ meaningful learning by examining the perspectives of students and SPs *during* the SP-student encounter. The research question was as follows: What are characteristics of meaningful learning moments for students during SP-student encounters and what are the perceptions of SPs during these moments?

**Methods:**

Twelve second-year Technical Medicine students conducted a medical consultation with SP presenting the same patient case. Each consultation was followed by qualitative, video-stimulated recall (SR) sessions, first with the student and then with the SP. During these sessions, students were prompted to articulate the thoughts they had during the consultation to identify meaningful learning moments. Video-fragments of the meaningful learning moments identified by the student were subsequently shown to the SP to explore their perceptions. All verbatim-transcribed recall data were thematically analyzed.

**Results:**

Student-identified meaningful learning moments were characterized by experiences that offered new insights, evoked emotions, and/or involved feedback-in-action from the SP. The SP’s perspectives of the same moments were compared to those of the students, revealing that these perspectives aligned approximately as often as they differed. SP experienced the moments as if they were the actual patient, fully embodying the patient’s role, while simultaneously maintaining an overview and being aware of the student’s learning position.

**Conclusions:**

This stimulated recall study enhanced our understanding of students’ learning during SP-student encounters. For students, meaningful learning moments involved new insights, emotional responses, and feedback-in-action from the SP. When SP fully engage in their patient role while maintaining awareness of the student’s learning context, they can respond authentically and supportively. Preparing SP to balance authentic role portrayal with educational awareness can enhance their contribution to students’ learning.

**Supplementary Information:**

The online version contains supplementary material available at 10.1186/s41077-025-00370-2.

## Background

Effective communication is essential in healthcare, as it significantly impacts patient outcomes, and the quality of care provided [1–5]. To enhance the communication skills of healthcare professionals, simulated patients (SPs) are commonly involved in medical educational programs [6, 7]. SPs are trained to portray patients with a specific condition to provide students with realistic practice scenarios and enable them to develop their communication skills [8]. The degree of standardization in these scenarios may vary depending on the learning context and objectives [6]. In contemporary undergraduate medical education programs globally, SPs play a significant role [9, 10].

Previous studies on SP feedback focused on the role of SPs’ feedback *after* SP-student encounters [9, 11–13], as this feedback is a crucial component of effective communication skills training [14]. From the learner’s perspective, feedback that reflects the patient’s experience is recommended and highly valued, as it helps learners better understand the patient’s viewpoint [13]. In addition to the use of feedback after the encounter, several studies aimed to unravel meaningful student learning *during* SP-student encounters [15–17], emphasizing the essential need to understand what students actually learn during these experiences. This is crucial because, according to the theory of experiential learning, learning is most effective when students actively engage in interactive moments [18].

In SP-student encounters, students immerse themselves in realistic clinical scenarios, allowing them to practice patient interaction in a safe environment. Experiential learning theory emphasizes that such experiences help students gain valuable insights, internalize knowledge, and build confidence [18–20]. Students reported that during their SP-student encounters, they gained insights into their communication skills based on the SPs’ reactions, described as implicit feedback-in-action [16]. Additionally, these SP-student encounters were perceived as contributing positively to their professional identity development [16]. SPs themselves indicated that they contributed to students’ meaningful learning by responding from multiple positions and perspectives during these encounters, including their assigned patient’s role, teaching aid position, and their personal experiences [15]. In this study, we understand meaningful learning as learning that is deeply connected to a learner’s existing knowledge structures, going beyond rote memorization. It involves actively making sense of new information by integrating it with prior knowledge and experiences [21, 22]. We also acknowledge students’ subjective interpretations in identifying which moments they experienced as meaningful.

Thus far, research into the difference in perspectives between students and SPs have not concentrated on the same moments within the same consultations. There has been limited exploration of the thoughts and experiences of both students and SPs *during* the same SP-student encounters [17]. The current study aims to bridge this gap by capturing the thoughts of both students and SPs during the same encounter, more specifically during *student-identified* meaningful learning (ML) moments in these encounters. This will deepen our understanding of how students learn communication skills and develop their professional identity while also clarifying the specific contributions of SPs during these interactions. In this way, educators can optimize the design of SP-based training, ensuring that students derive the maximum benefit from these experiences.

This study aims to explore the following research question: What are characteristics of student-identified ML moments during SP-student encounters and what are the perceptions of SPs during these moments? Capturing and measuring these learning experiences can be challenging, as it is not feasible to pause the encounter and query students about their learning processes in real time. Therefore, a stimulated recall (SR) study design was used [23–25].

## Methods

### Theoretical framework

An interpretivist research paradigm was adopted, along with a qualitative approach [26] using video-SR of communication training encounters with second-year Technical Medicine (TM) students. The study consecutively explored both the students’ and SPs’ perspectives on student-identified ML moments [24].

### Setting

This study was conducted at the University of Twente (Netherlands) undergraduate TM 3-year bachelor program. A technical physician is a licensed healthcare professional who contributes to optimizing patient care using technology [27]. Their role focuses on integrating technological innovations into direct patient care and is legally licensed to independently perform medical consultations and some medical procedures. Technical physicians have the knowledge and experience to tailor technology-based medical procedures to the individual patient [27]. Therefore, learning medical communication skills is essential in their training. The communication program includes 15 SP-student encounters per student throughout the 3-year bachelor curriculum. These encounters allow students to practice basic communication skills, such as listening, summarizing, and asking open questions, and advanced communication skills, such as breaking bad news.

SPs are individuals who play a crucial role in the training and assessment of communication skills by realistically and authentically portraying a patient case, while also providing feedback from the patient’s perspective [28]. An SP can be a layperson, a trained actor, or someone from another background; they are always trained to perform a role and provide feedback. The 40 SPs at the University of Twente are laypeople, vary in SP experience from 1 to 20 years, work on a non-contract basis, and range in age from 30 to 70 years, with a male/female ratio of 30/70. SPs receive biannual training in role-play and feedback skills supplemented four annual role-specific sessions for assessment scenarios. Training emphasizes acting techniques such as emotional expression, consistency, and credibility. Sessions are conducted in groups of 4 to 20 participants, depending on the training objectives. Two professional training actors and an SP educator facilitate these sessions. The majority of SPs are involved in SP-student encounters more than 15 times annually.

### Participants

All 124 s-year students (36% male/64% female) enrolled in the communication course were informed by email about the study. All these students had prior experience with SP-encounters, having participated in approximately 8 to 10 SP consultations during their first year and the early part of their second year. In total, 12 students and 12 SPs participated voluntarily in the research session. This sample size was intentional, based on the expectation that the data would be rich and in-depth. With each student-SP pair participating in separated stimulated recall session, this approach yielded a total of 24 stimulated recall sessions. The inclusion of students took place based on the order of registration (1 male/11 female, age range 18–21). Students could either consider the SP-encounter prior to the stimulated recall session as extra practice or as a substitute for regular practice. They all chose to treat the session as extra practice. The researchers were not part of the assessment team evaluating the students enrolled in the study.

The SPs (3 male/9 female, age 36–76, years of experience as SP 3–17) were assigned to the scenario based on their availability and suitability for this case, just as for regular education sessions. Written and voluntary informed consent was obtained from all participants.

### Data collection

**Fig. 1:**
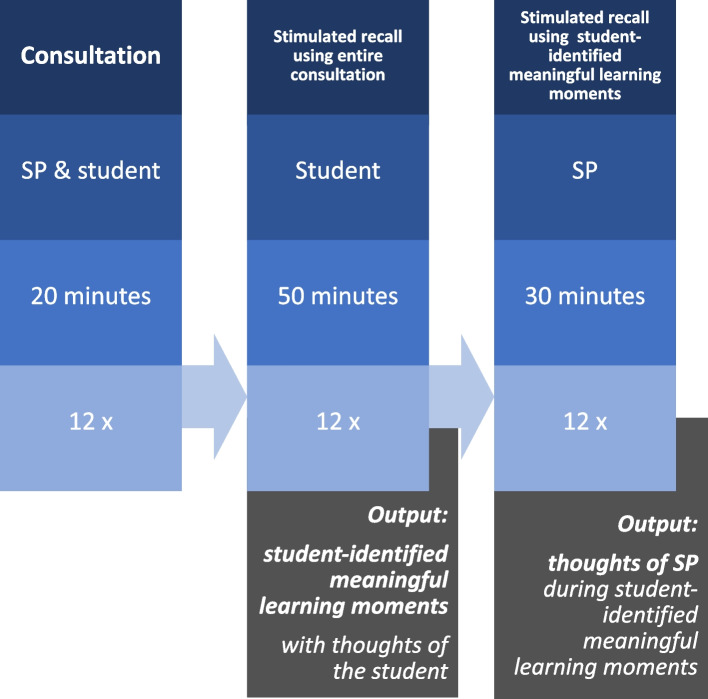
Data collection

The scenario involved a 20-min consultation with an SP, including physical examination (Fig. 1). No other students were present during the consultation and there was no feedback from the SP afterwards. The case for the scenario was an incidentally discovered aortic aneurysm, focusing on communication skills rather than the routine of taking a medical history. In this case, the focus is not diagnostic reasoning, but on management reasoning, a topic not previously covered in their education, which places students outside of their comfort zone [29]. The student-SP consultations were recorded using CAE LearningSpace Enterprise [30]. The recorded consultations themselves were not used as research data but served as input to guide the stimulated recall sessions.

#### Stimulated recall

Stimulated recall (SR) is a method in which participants are prompted to reflect on their thoughts during a prior activity, using video recordings as cues [24]. In our study, we used the recorded consultations as the primary input for the stimulated recall sessions. We developed separate SR protocols for the SR sessions with students (Appendix 1), and for the SR sessions with SPs (Appendix 2), and an overall protocol for the workflow (Appendix 3). The three protocols were refined based on a pilot SR session. Four researchers, consisting of two of the authors (AL, MG), a colleague of the authors who was a lecturer in the course, and an intern, conducted the stimulated recall sessions.

#### SR session with student

Directly after every encounter, a video-SR session with the student took place, during which the student was prompted to articulate the thoughts the student had during the consultation (Fig. 1). These SR sessions were audio-recorded. The researcher watched the recording with the student and asked the student to verbalize as openly as possible all thoughts they had during the consultation. This session lasted up to 50 min. Each time the student verbalized a thought, the researcher asked standardized follow-up questions to identify learning moments such as follows: “What were you thinking? What were you doing? What were you feeling? Did you learn anything from this moment? If so, what was it? Will this moment stick with you? Is it a ML moment?” No definition of what constituted a ML moment was provided to the students, as the identification of such moments was deliberately left to their individual judgment. Each time the student indicated that it was a ML moment, the moment was marked and numbered. A facilitator made notes and recorded the times a student reported a moment to be a ML moment.

#### SR session with SP

After the session with the student, the SR session with the SP followed. This session lasted up to 30 min (Fig. 1) and was audio-recorded. The SP had not had contact with other SPs or students in the meantime. The student-identified ML moments were presented to the SPs, with a request to verbalize their thoughts as openly as possible. During the SR sessions, the SPs were unaware that the moments shown to them were ML moments for the students. This approach was intentionally taken to avoid introducing any bias. During the SR with the SP, the researcher asked the following standardized follow-up questions: “What were you thinking? What were you feeling? What were you doing? Was that deliberate or unintentional?” Sometimes, further questions were asked to clarify from which perspective the SPs was verbalizing thoughts.

### Analysis

The transcribed data from the SR sessions with the students and the SPs were analyzed using thematic analysis [31]. Thematic analysis allows us to systematically analyze and interpret patterns of themes within the data, exploring diverse perspectives [32, 33]. For each encounter, an overview of the learning moments was created to map the learning moments and thoughts expressed by both the student and the SP. The overviews were created by one researcher (AL) and every overview was checked by one other researcher (JR, AvN, MG).

Although this overview introduced some structure, the thematic analysis itself was primarily inductive, as no predefined coding framework was used. These overviews for each encounter, with the thoughts of the student and SP, were subsequently coded by one researcher (AL) and checked by other researchers (AvN, MG), supported by the Atlas.ti. software program, to categorize the content and identify themes. For each ML moment, we first examined whether the interpretations of the student and the SPs were aligned. Following this comparative step, we adopted a thematic approach to identify overarching patterns across all reported experiences. This broader perspective allowed us to uncover themes that were meaningful for each group as whole, reflecting how students and SPs engage with and make sense of student-identified ML moments. Data were reviewed jointly, followed by collaborative discussion among all researchers about the appropriateness of the codes and themes.

### Reflexivity and research team

All authors adopted a reflexive attitude to discuss their initial observations of the data concerning their own interests and biases [33, 34]. All researchers discussed reflexivity to identify their potential biases and presuppositions. They considered their own occupational roles and how these might affect their initial reading of the data. In the spirit of reflexivity, we provide the authors’ relevant backgrounds. Researcher AL is a lecturer and SP educator at the Department of TM with 15 years of experience working with SPs. MG is a lecturer and researcher at the Department of TM with substantial experience in human and non-human simulation education. AvdN has extensive research experience using qualitative methodologies in the field of medical education. JJR is a professor in the field of human simulation and has worked with SPs since 1985. WvM is a clinician, with a PhD in medical education with ample experience in qualitative research, who supervises PhD candidates, e.g., in different aspects of healthcare simulation.

### Ethical approval

This study was ethically approved by the BMS Ethics Committee of the University of Twente (number 211143).

## Results

Twenty-four SR sessions resulted in a total of 83 student-identified ML moments, capturing both students’ and SPs’ perspectives. First, we provide an overview of the different aspects of student-identified ML moments, followed by an in-depth exploration of these moments from both the students’ and SPs’ perspectives.

### Meaningful learning moments

From their simulated patients’ consultations, the 12 students identified a total of 83 moments that they personally marked as ML moments during the stimulated recall sessions, as shown in Table 1. On average, each student identified 7 ML moments, with a minimum of 4 and a maximum of 13 moments per student. Student identified ML moments as those characterized by experiences that offered new insights (*This is new to me!*), evoked emotions (*I feel…*), and/or involved feedback-in-action from the SP.

**Table 1:** Meaningful learning (ML) moments

Data	Number
Total ML moments identified by 12 students	83
Average ML moments per student	7
Minimum ML moments	4
Maximum ML moments	13
ML moments responded to by SPs	83 (100%)
Aligned	42
Different perspectives	41
**ML moment identified by students**	**Frequency**
"This is new to me!”	23
“I feel…”	52
Feedback-in-action	35
**SP experience during ML**	**Frequency**
“I’m the patient”	50
“I see what the student is doing”	46

**Fig. 2:**
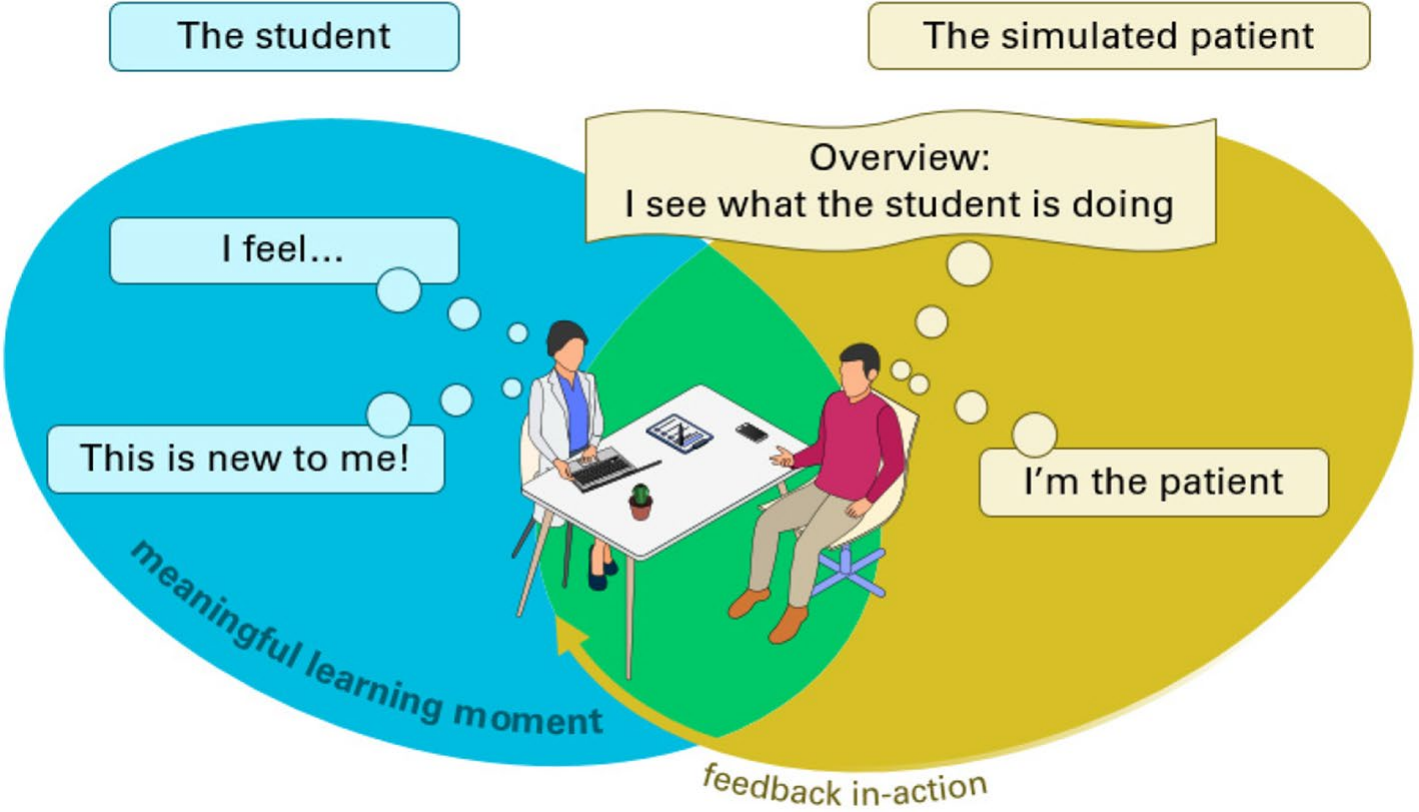
Representation of a student-identified meaningful learning moment

The SPs responded to all the 83 shown student-identified ML moments. In half of the moments, the interpretation of the learning moment identified by the student and the simulated patient (SP) aligned. In the other moments, the SP had different perspectives during the moment, reflecting a different interpretation of the essence of the moment. SPs experienced the moment as being the patient (*I’m the patient*) while simultaneously having an overview and being aware of the student’s learning position (*Overview: I see what the student is doing*). Figure 2 visually represents these identified characteristics of ML moments during an SP-student encounter. The two ovals in Fig. 2 do not fully overlap because of the different perspectives of the student and the SP.

Examples 1 and 2 illustrate how the various characteristics of student-identified ML moments were expressed through the thoughts of the students and SPs, offering a holistic view of the interplay between their perspectives.

In Example 1, from encounter 4, ML moment 4 illustrates how the student experienced a new and confusing situation, while the SP perceived the same situation differently. The discrepancy in perception between the student and the SP might not negatively impact learning, as students still regarded these moments as ML moments.

#### Example 1

Non-alignment of student and SP perspectives.

**Table:** 

Student: The patient started to partially undress on the chair (feedback-in-action). That’s when I went into a mini panic. It’s very important to me that the patient can choose whether they undress or not. I pay close attention to what I say in those situations. Maybe I forgot to mention that undressing should be done in the changing room, or it was misunderstood. It was a new confusing situation that triggered a little panic.
SP: The student indicated what to expect during the physical examination. Much more spontaneous than the rest of the consultation (overview). I found it much more natural, she did it neatly. I knew what to do. I went along with it and I did what she said (I’m the patient). There was more interaction here.
Encounter 4, ML 4

Example 2, from encounter 2, ML moment 4, illustrates how the student experienced feedback-in-action from the SP, who was aware of the learning situation while simultaneously embodying the patient role.

#### Example 2

Alignment of student and SP perspectives.

**Table:** 

Student: The patient doesn’t understand at all what I’m saying (feedback-in-action). This may be very vague language for the patient while it’s very logical for us. I realized that what is logical for me may not be for the patient.
SP: The student mentions the word “circumstances.” I don’t understand that question. I shake my head and look somewhat offended. The use of language is something from theory, she could do it differently. I also think, she's still a student. I play on that to challenge her to articulate herself better so she can learn from it. I feel confused in my role. As myself, I feel that I’m consciously engaged. I respond to the word “circumstances” very consciously.
Encounter 2, ML 4

We will explore these different characteristics of ML moments in depth below. First, the students’ perspective is explored; second, that of the SPs. We provide context by presenting illustrative quotes about different ML moments (ML#).

### The student perspective

#### This is new to me!

The ML moments identified by students offered new experiences, as described by this student:

*I didn’t know what to say, the patient had no symptoms. Normally, you see a patient because the patient is having symptoms. Not this time. I thought, okay, I’m not sure how to deal with this exactly. Something to dive into and look up at home. What do you do if someone isn’t in pain, but it’s still something fairly serious?*

*(Student, Encounter 3, ML 4)*

Sometimes, students were confronted with an unexpected situation:

*The patient is very agreeable. He immediately says ‘fine’ to the treatment. I was surprised. I still need to try to provide more information. This went very smoothly. Does he just want to cut the conversation short? Am I taking too long or something? Usually, more discussion is needed. That wasn’t the case this time, but I still want to provide more information.*

*(Student, Encounter 5, ML 9)*

#### I feel….

Students described various emotions during ML moments such as discomfort, uncertainty, positivity, and confusion. In the next example, the student was performing a physical examination and experienced discomfort.

*The patient is making jokes, which is nice for the atmosphere. But I did think it might come across as if I don’t know what a heartbeat is and why I need to listen. I could have provided more explanation. It feels uncomfortable.*

*(Student, Encounter 7, ML 9)*

Positive feelings were also associated with ML moments. For example, when the student experienced success.

*The question ‘how did that affect you?’ was just answered by the patient. I was glad she understood that question well. […] I’m glad it worked out. It was an important learning moment because previously it sometimes went wrong. And maybe I only see the mistakes as important or something.*

*(Student, Encounter 8, ML 3)*

#### Feedback-in-action

Students experienced feedback-in-action across various learning moments, regardless of whether the SPs interpreted the moment in the same way and whether the SPs was giving feedback-in-action intentionally or not, as the next example shows:

*The patient asked what I said; I speak a bit softly. It is a learning moment because it’s sort of a reminder to start consultations louder with others.*

*(Student, Encounter 4, ML 1)*

### The SP perspective

#### I am the patient

SPs’ utterances indicated that they fully immersed in their roles. SPs indicated that they mostly reacted from the patient perspective.

*I’m sitting there as a patient thinking, ‘Well, I just want this to be over.’ That’s what I’m thinking. I’ve said that I have no physical symptoms. I’ve unintentionally registered that yes, I must sit here because you want to discuss it, but I didn’t really want to be here at all.*

*(SP, Encounter 5, ML 1)*

The following quote also illustrates how the SPs is fully immersed in the patient role, feeling the patient’s emotions:

*Yeah, good, I appreciated that she asked about it. I was happy that I could tell her that I was doing something good for my health. Because I was aware of that.*

*(SP, Encounter 11, ML 8)*

#### Overview: I see what the student is doing

While SPs indicated that they primarily act as the patient during the encounter, they are simultaneously aware of the (learning) situation, having an overview of the situation.

*Measuring blood pressure looks very clumsy and awkward. She’s very nervous. If you do it like this in real life, it just won’t do. She needs a lot of practice. Then I speak from myself, I come from that world. I think she must do it anyway, so I’ll just let it be. I’m not going to confront her or anything. As a patient, I’m not the most difficult, quite easy-going. So, I think let her carry on.*

*(SP, Encounter 5, ML 7)*

Having an overview of the situation makes it possible for SPs to choose not to intervene during the encounter, allowing the situation to unfold for the student.

*I thought, do I have to tell you everything three times, why don’t you know that this is not new to me, but that I’ve had it for 7 years. […] It annoyed me that she didn’t know, I felt that she should know. […] I started to talk a bit irritated, arms crossed and started to look at her more like ‘are you serious?’ With a 3rd-year student, I would probably have been angry, but I consciously didn’t do that here. I had thought about it at home. That’s why I keep talking and give her everything she wants to know. But here and there, I do notice that it’s annoying.*

*(SP, Encounter 12, ML 2)*

## Discussion

This study aims to explore the characteristics of meaningful learning (ML) moments for students during SP-student encounters, as well as the perceptions of SPs during these moments. By analyzing student-identified ML moments alongside SPs’ perceptions, this study enhanced our understanding of student learning during SP-student encounters.

The findings indicate that student-identified ML moments are closely linked to the student’s subjective experience of the interaction. Students frequently described these moments as instances where they encountered new insights, experienced emotional engagement, or received immediate feedback from the SPs. These moments appear to be highly personal and embedded in the immediacy of the consultation. Moreover, the SP’s ability to fully embody the patient role, while maintaining awareness of the educational context, seems essential for facilitating such learning. Four key findings will be discussed in more detail below: the alignment or non-alignment between students and SPs’ perspectives, the students’ emotional experience as characteristic of ML, the SP’s embodiment of the patient role, and the SP’s ability to switch between positions.

### Alignment (or non-alignment) between student and SP perspectives

The SPs’ perceptions of the student-identified ML moments either aligned closely with the students’ view, as illustrated by the green overlap in Fig. 2, or differed, as indicated by the yellow oval. These discrepancies did not appear to hinder student learning, as students still identified these moments as ML moments. In addition, only the student can determine whether something constitutes a meaningful learning moment for themselves. Even though meaningful learning is thus ultimately defined by the student, exploring the differences in how students and SPs experience the same moments can yield valuable insights. The observed discrepancy in perceptions between students and SPs aligns with findings from Laughey et al. (2018), who highlighted SPs’ focus on elements such as active listening, empathy, human connection, and the flow of information during consultations [35]. In contrast, Hulsman and van der Vloodt (2015) showed that students tend to focus on technical aspects such as structuring the consultation [36]. Our study underscores the value SPs bring in recognizing interpersonal dynamics, which may otherwise be overlooked by students focused on task completion [35]. In the following discussion, we further explore the different experiences of students and SPs.

### Emotional experience as characteristic of meaningful learning

The second key finding highlights the critical role of students’ emotional experiences in their learning and development. ML moments often reflected novelty or impact, aligning with Vygotsky’s zone of proximal development (ZPD). The ZPD represents the space between what learners have mastered and the new skills or knowledge they are ready to acquire [37]. Challenges at the edge of the ZPD can act as catalysts for growth [37]. Emotions impact students’ learning experiences, influencing attention, memory, problem-solving, and motivation as noted by LeBlanc and Posner [38]. Emotional responses at the edge of the ZPD are essential for ML, stimulating reflection [22]. Our findings support this by emphasizing the importance of emotions in learning, highlighting the need for simulations that challenge the ZPD and intentionally engage students emotionally to foster reflection and support ML.

### SP perceptions during meaningful learning

#### Embodiment of the patient role

One key aspect of being an SP is the ability to embody the role of the patient fully and, in that way, contribute to student-identified ML moments. This requires SPs to embody the perspective of a real patient, not just in terms of acting but in conveying emotions, behaviors, and responses authentically. Stanislavski (1863–1938), one of the first modern acting teachers, developed an approach for acting, often referred to as “The System” [39–42]. This theory is highly relevant for SPs, highlighting the link between internal emotions and external behaviors to ensure authentic, empathetic, and patient-centered performances [41]. By adopting Stanislavski’s concept of the *state of* “*I am*,” SPs can immerse themselves in the patient situation [39, 40]. In addition to the work by Smith et al. [42], our current study shows that Stanislavski’s theories are not only highly applicable to the SPs’ acting work but also to creating optimal learning moments for students. By embodying the role, SPs facilitate students’ ML processes. Techniques from the acting world can help SPs enhance their skill, suggesting the practical implication of incorporating additional training in acting methodologies. Instructors might need to rethink SP preparation, integrating principles from acting theory to ensure that SPs can embody their role.

#### Switching positions

Maintaining a helicopter view is also important for SPs to effectively respond to students’ learning needs. This is similar to the work of Smith et al. [42], who discussed circles of concentration: each SP, trainee, and facilitator has small, medium, and large circles of concentration around them [42]. This explains how SPs stay focused on the role while maintaining awareness in the simulation. As the SPs and student interact, they move from private circles to shared ones, allowing the SPs to adapt to the student’s needs and better support student’s learning [42]. The SPs’ ability to balance immersion in the patient role with maintaining an observational stance toward the student is crucial for creating ML moments. The findings of the current study suggest that by adopting a helicopter view SPs can observe students’ learning, identify struggles, and exercise restraint in intervening, sometimes allowing the scenario to unfold naturally. Such a non-intervention decision may strategically give students space to self-discover, reflect, and learn from (near) mistakes [43]. The current study builds on previous research that explores the different positions of SPs, including the use of positioning theory by Sargeant et al. [44], the concept of Multiple Personas by Sullivan et al. [45], and the characterization of SPs’ dual role as both patient and teaching aid by Lovink et al. [15]. In addition to previous studies, this current research shows that SPs’ ability to maintain a helicopter view while staying immersed in their role is crucial for facilitating ML moments.

### Challenges and strengths

A strength of this study is the use of in-depth video-SR sessions to investigate the thoughts of both the student and SP during SP-student encounters, rather than interview studies where participants must recall information from memory. The SR method provides the closest approximation to capturing the real-time thoughts participants had during the consultation. However, watching the video of the consultation may itself trigger a reflective process. As a result, we cannot be entirely certain that the thoughts expressed are exclusively those experienced during the consultation; they may also include reflections formed afterward. This study is based on a sample from one technical medical school. However, by describing insights from both the students and SPs, we provide a comprehensive perspective on the subject and enhance credibility. We promote transferability by describing the findings and the context in detail and discussing our findings with existing literature from different settings.

## Conclusion

This study indicates that SPs do not need to identify which consultation situations are most likely to lead to ML moments for students. Instead, the occurrence of ML moments is more strongly associated with how students experience the interaction. From the student perspective, ML moments were often characterized by the experience of something new, by the evocation of emotions, and/or by receiving feedback-in-action from the SPs. These elements suggest that meaningful learning is personal, rooted in the student’s unique experience and emotional engagement, and embedded in the immediacy of the consultation. For such moments to occur, it is crucial that SPs fully immerse in their role as patients, engaging with students from the patient perspective while remaining aware of the students’ learning context and having an overview of the situation. This dual awareness enables SPs to respond naturally and responsively, without needing to control or predict learning outcomes. This study encourages us to better prepare our SPs for their task as SPs. When they feel comfortable in their role and are trained to have an overview of the students’ learning situation, SPs can be given the freedom to act and react according to how they feel as the situation unfolds, within the boundaries of the educational goals and the script, thereby optimizing students’ learning potential.

## Supplementary Information

Supplementary Material 1.

Supplementary Material 2.

Supplementary Material 3.

## Data Availability

The datasets used and analyzed during the current study are available from the corresponding author on reasonable request. Due to the sensitive nature of the data and the small participant population, full availability of the data is not possible in order to protect participant confidentiality and minimize the risk of identification.

## Abbreviations

SP: Simulated patient
ML: Meaningful learning
TM: Technical medicine
SR: Stimulated recall
ZPD: Zone of proximal development
