# Supplementary material for: Learning in the moment: simulated patients’ engagement in students’ meaningful learning during communication training—a stimulated recall study
Source: Adv Simul (Lond). 2025 Sep 26;10:46. doi: 10.1186/s41077-025-00370-2 (PMC12465989; doi:10.1186/s41077-025-00370-2)
Supplement: Supplementary file 1 — Supplementary Material 1. [file 41077_2025_370_MOESM1_ESM.docx]

**APPENDIX 1: Protocol SR Student**

**Introduction before stimulated recall**

First of all, I would like to sincerely thank you for participating in this research. I will now briefly review the information from the information letter with you.

**Purpose of the research:**

Simulation-based education with simulated patients is an important part of communication training in various medical programs, including Technical Medicine at the University of Twente. To better understand how this form of education contributes to student learning, a study has been initiated. The research focuses on student learning during consultations and how simulated patients contribute to this process.

**Stimulated recall procedure:**

During the stimulated recall, we will watch the recording of the consultation together. We are interested in hearing what you were thinking during the consultation. I will pause the video at your request, so you can share your thoughts. Please raise your hand if you want me to pause the video. There are no wrong answers or comments. All the thoughts you had during the consultation may be valuable for the research. We would appreciate it if you could share your thoughts as openly as possible. Anything that comes to mind is fine! My colleague will be present to take notes and record times. It is possible that we won’t go through the entire consultation, which is okay.

**Research vs. teaching:**

This stimulated recall is entirely separate from your performance in the VCPG course, so it will not affect your assessment of communication skills. I am here as a researcher, not as a teacher, and I will not assess your performance.

**Privacy:**

The conversation will be recorded with a voice recorder and transcribed. The data will be processed confidentially and anonymously and will only be used for research purposes. The data will be kept for 10 years. Participation in the study is entirely voluntary. You may withdraw from the study at any time without providing a reason.

Have you been adequately informed? Do you have any questions?

**Would you please sign the consent form?**

**General questions**

Participant number:

Age:

Gender:

Year of study: 2

Date:

Now, let's move on to the actual research. We will begin with a warm-up.

**1. Warm-up**

Instruction: Please read the case information ‘TM-info’ again. What do you think when you read this? Try to express your thoughts aloud.

If someone doesn’t verbalize any thoughts after reading, then ask: What do you think now?

After the warm-up: Now you have an idea of what it’s like to express your thoughts. Are you ready to watch your consultation? Do you have any questions?

**Start stimulated recall and START voice recorder**

**2. Video-stimulated recall**

Instruction: We will now review your consultation. We are very interested in what you were thinking during the consultation. I ask you to share any thoughts you had during the consultation that you still remember. If you want me to pause the video, just raise your hand. Then, share your thoughts openly about what you were thinking at that moment during the consultation. Again, there are no wrong answers!

Are you ready?

If the student has not said anything after 3 minutes: Don’t forget to express what you were thinking during the consultation. If you raise your hand, I will stop the video.

If the student remains silent, remind them every 3 minutes: "Are you remembering to verbalize your thoughts?"

**The following questions should be asked whenever the student starts verbalizing thoughts (when the video is paused):**

1. What were your thoughts at this moment in the consultation? (not always necessary, sometimes clear from the student’s description)
2. What were you doing at this moment in the consultation?
3. What were you feeling at this moment in the consultation?
4. Did you learn from this moment? If so, what exactly?
5. Do you think this moment will stay with you?
6. Would you describe this moment as an important learning moment? (This question is similar to question 4 but slightly different.)

**Conclusion**

- Are there any thoughts that have come to mind now that you would like to share?
- Is there anything else you would like to say on this topic?
- Would you like to be kept informed? (If yes, please note the email address on the informed consent form.)
- Thank you very much for your cooperation.

**STOP voice recorder**
