# Supplementary material for: Learning in the moment: simulated patients’ engagement in students’ meaningful learning during communication training—a stimulated recall study
Source: Adv Simul (Lond). 2025 Sep 26;10:46. doi: 10.1186/s41077-025-00370-2 (PMC12465989; doi:10.1186/s41077-025-00370-2)
Supplement: Supplementary file 2 — Supplementary Material 2. [file 41077_2025_370_MOESM2_ESM.docx]

**APPENDIX 2: Protocol SR Simulated Patient**

**Introduction before stimulated recall**

First of all, I would like to sincerely thank you for participating in this research. I will now briefly review the information from the information letter with you.

**Purpose of the research:**

Simulation-based education with simulated patients is an important part of communication training in various medical programs, including Technical Medicine at the University of Twente. To gain more insight into how this form of education actually contributes to student learning, a study has been initiated. The research focuses on student learning during consultations and how simulated patients contribute to this process.

**Stimulated recall procedure:**

For the stimulated recall, we will watch the recording of parts of the consultation together. I will pause the video at your request so that you can express your thoughts. Please raise your hand if you want me to stop the video. There are no wrong answers or comments. Any thoughts you had during the consultation may be valuable for the research. We would appreciate it if you could share your thoughts as openly as possible. Everything is fine!

**Research vs. teaching:**

The stimulated recall is separate from your role as a simulated patient, which means that it will not affect the hours you are available as a simulated patient. I am here in my role as a researcher.

**Privacy:**

The interview will be recorded with a voice recorder and transcribed. The data will be processed confidentially and anonymously and will only be used for research purposes. The data will be kept for 10 years. Participation in the study is entirely voluntary. You may withdraw from the study at any time without providing a reason.

Have you been adequately informed? Do you have any questions?

**Would you please sign the consent form?**

**General questions**

Participant number:

Age:

Number of years as a simulated patient at the University of Twente:

Gender:

Year of study: 2

Date:

Now, let’s move on to the actual research. We will begin with a warm-up.

**1. Warm-up**

Instruction: Please read the case information 'information spontaneously' again. What do you think when you read this? Try to express your thoughts aloud.

If someone does not verbalize any thoughts after reading, use the following prompt:

“Try to verbalize your thoughts. What do you think now?”

After the warm-up: Now you have an idea of what it’s like to verbalize your thoughts. Are you ready to watch the consultation? Do you have any questions**?**

**Start stimulated recall and START the voice recorder**

**2. Video-stimulated recall**

Instruction: We will now review parts of the consultation. It may be that we do not go through the consultation chronologically (Note: start with the key learning moments). I can explain how we selected these moments later. If you want to verbalize your thoughts, raise your hand, and I will pause the video. Then, share as openly as possible what you were thinking at that moment. Are you ready?

We will now look at moment 1. Please share what you were thinking. Again: everything is fine!

If the SP asks whether they should respond from the role or as themselves, you can reply: “What were your thoughts at that moment?”

- If the SP does not say anything after viewing the moment: “What were your thoughts at that moment?”
- If the moment lasts more than 1 minute, remind them after 1 minute: “I’m really curious about what you were thinking at this moment in the consultation.”
- If the entire consultation is shown because the student did not identify any learning moments, then remind them every 3 minutes: “Don’t forget to verbalize your thoughts during the consultation. If you raise your hand, I will stop the video.”

**Questions to deepen the reflection at a key learning moment (after the SP has verbalized their thoughts):**

1. What were you thinking at this moment in the consultation?
2. What were you feeling at that moment?
3. What were you doing at that moment?
4. Was that conscious or unconscious?
5. Repeat this for the other key learning moments identified by the student. Always refer to the number of the learning moment: “Now let’s look at moment X…”

**Conclusion**

- Are there any thoughts that come to mind now that you would like to share?
- Is there anything else you would like to add on this topic?
- Would you like to be kept informed about the research? (If yes, please provide your email address on the informed consent form.)
- Thank you very much for your cooperation!

**STOP the voice recorder**

If needed, explain how the choice of moments was made.
