# Supplementary material for: Learning in the moment: simulated patients’ engagement in students’ meaningful learning during communication training—a stimulated recall study
Source: Adv Simul (Lond). 2025 Sep 26;10:46. doi: 10.1186/s41077-025-00370-2 (PMC12465989; doi:10.1186/s41077-025-00370-2)
Supplement: Supplementary file 3 — Supplementary Material 3. [file 41077_2025_370_MOESM3_ESM.docx]

**APPENDIX 3: Protocol SR General**

**Requirements per researcher:**

- Laptop with power source and Windows Voice Recorder
- Computer in the research room to record and play back consultations
- 6x informed consent forms, per researcher
- Timer on your phone
- 3x Protocol SR student
- 3x Protocol SR SP
- Case and TG information for the warm-up
- 3x form to track learning moments
- 3x general protocol
- Schedule

**Step-by-step plan per session**

(Bold text means: by the assistant)

1. Welcome the student and SP in the hallway outside the consultation room.
2. The student can enter the consultation room. The SP waits in the hallway.
3. Start the LS recording of the consultation under 22-11-29 stimulated recall and add the names of the student and SP.
4. Indicate that the student can begin the consultation.
5. Discuss with the research assistant their tasks:
   a) Record the times in LS
   b) Provide a description of the moment
   c) Note if it’s a key learning moment and number the learning moments
   d) Record the time in the Windows voice recorder if it's a key learning moment
   e) Time management: 50 minutes before the session with the student
6. The consultation can last up to 20 minutes. After 20 minutes, knock on the door and allow 2 more minutes for a wrap-up.
7. Stop the recording once the consultation has ended.
8. Invite the student to come to the research room. The SP can remain in the consultation room. There will be coffee. The SP should not talk to other SPs, so they remain in the consultation room.
9. **Start the timer for 50 minutes.**
10. Read the introductory points to the student.
11. Confirm everything is clear.
12. Request informed consent and have the student sign the form. Ensure the name is filled in. Also, sign the form yourself.
13. Fill in the participant number (see schedule), age, gender, and date on the SR Student protocol.
14. Begin the warm-up.
15. Check if everything is clear.
16. Set up the relevant consultation on the computer in the research room in LS.
    If the audio and video in LS are not synchronized, you can go back to the homepage of LS and restart the consultation (max 2 times).
17. **Start the voice recorder!**
18. Begin the actual research according to the SR Student protocol.
19. **The end time for the stimulated recall is indicated in the schedule. You may only go through part of the consultation. This is not a problem.**
20. **Every time the student indicates that the video can be stopped, record the time from LS on the overview sheet on the table.**
21. **After questioning according to the SR Student protocol, note whether this moment is considered a key learning moment (yes or no).**
22. **Every time the student indicates that it is a key learning moment, record the time of the Windows voice recorder.**
23. **Stop the voice recorder!**
24. Thank the student. The student can leave the research room. They will not receive feedback on the consultation.
25. Save the recording under the participant number + the addition that it’s for the student.
26. Review the notes with the assistant to ensure everything is clear. Thank the assistant.
27. Set up the first learning moment in LS. If the student hasn’t identified key learning moments, use the moments when the student verbalized their thoughts. If the student did not verbalize any thoughts, show the entire consultation. If the number of key learning moments is limited, start by showing those moments and then move on to the other moments.
    Start the learning moment 5 seconds before the indicated time. Stop the video when the learning moment described by the student ends. This often lasts only a few seconds (tens of seconds).
28. Invite the SP to the research room.
29. Set the timer for 30 minutes.
30. Read the introductory points to the SP.
31. Confirm everything is clear.
32. Request informed consent and have the SP sign the form. Ensure the name is filled in. Also, sign the form yourself.
33. Fill in the participant number (see schedule), age, gender, date, and number of years of experience on the SR SP protocol.
34. Start the warm-up.
35. Check if everything is clear.
36. **Start the voice recorder!**
37. Begin the actual research according to the SR SP protocol. Mention the number of the moment each time.
38. The end time for the stimulated recall is indicated in the schedule.
39. **Stop the voice recorder!**
40. Thank the simulated patient.
41. Save the recording under the participant number + the addition that it’s for the SP.
